# Supplementary material for: A data-driven network decomposition of the temporal, spatial, and spectral dynamics underpinning visual-verbal working memory processes
Source: Commun Biol. 2023 Oct 23;6:1079. doi: 10.1038/s42003-023-05448-z (PMC10593846; doi:10.1038/s42003-023-05448-z)
Supplement: Supplementary file 2 — Supplementary materials [file 42003_2023_5448_MOESM2_ESM.pdf]

Supplementary Materials

A data-driven network decomposition of the temporal, spatial, and spectral dynamics underpinning visual-verbal working memory processes

Chiara Rossi[1,2,*], Diego Vidaurre[3], Lars Costers [1,4], Fahimeh Akbarian[1,2], Mark Woolrich[5], Guy Nagels[1,6,7], Jeroen Van Schependom[1,2,*]

[1] AIMS lab, Center for Neurosciences, Vrije Universiteit Brussel, Brussels, Belgium
[2]Department of Electronics and Informatics (ETRO), Vrije Universiteit Brussel, Brussels, Belgium
[3] FNIRS, Aarhus university, Aarhus, Denmark
[4] icometrix, Leuven, Belgium
[5] OHBA, University of Oxford, Oxford, United Kindom
[6] UZ Brussel, Department of Neurology, Brussels, Belgium
[7] St Edmund Hall, University of Oxford, Oxford, United Kindom

[*]corresponding authors chiara.rossi@vub.be jeroen.van.schependom@vub.be

1. Reaction time and Accuracy of response

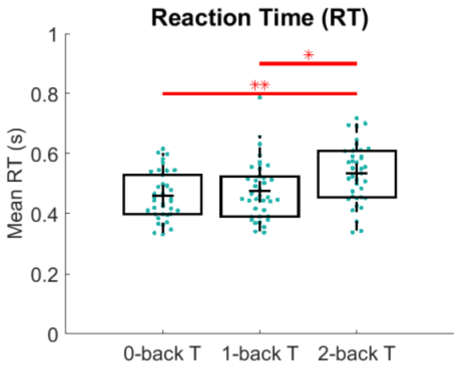

Figure S1 Distribution of mean reaction times (RTs) between the three paradigm target conditions (0-back, 1-back, 2-back). The mean RTs for the 2-back is significantly increased compared to the 0-back and the 1-back conditions. (Wilcoxon rank-sum test, *0.005< p <0.05, **p <0.005)

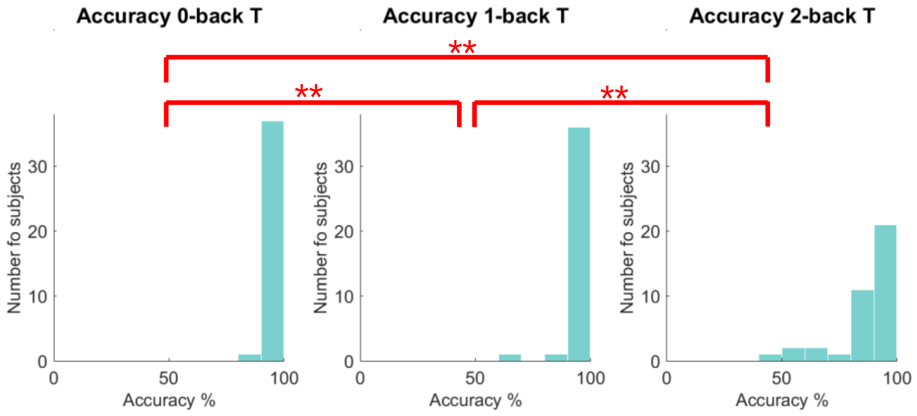

Figure S2 Distribution of accuracy of response for the three target conditions (0-back, 1-back, 2-back). The accuracy of response decreases significantly when increasing the WM load, from 0-back to 2-back conditions. (Wilcoxon rank-sum test, *0.005< p _value<0.05, **p_value <0.005)

The mean reaction times (RTs), as the time between the stimulus onset and the button press, are 458.7±79.0 ms for 0-back T, 477.0 ±96.4 ms for 1-back T, and 534.4±98.0 ms for 2-back T. The mean RTs increase with increasing WM load (0, 1, 2-back T). In particular, the mean RTs during the 0-back T are significantly faster than the 2-back T (Wilcoxon rank-sum test, p_value<0.005) and the same hold for the 1-back T as compared to the 2-back T (Wilcoxon rank-sum test, p_value<0.05). The increasing reaction time in parallel with the increased working memory load is often reported in the WM literature and reflects the increasing task difficulty. Additionally, we explored the worsening task performance considering the response accuracy, defined as the number of correct answers over the total target trials for the specific task condition. We computed the accuracy for the 0-back T 99.3±3.3 %, for the 1-back T 97.0±6.9 %, and for the 2-back T 87.8±14.5 %. The accuracy of response decreases significantly with the increasing WM load (Wilcoxon rank-sum test, p_value<0.05).

2. Inference with 12 states

Figure S10 reports the results for the 12 states inference. From the event-related (ER) analysis of the states' posterior probabilities of activation, we identify 6 states with an evoked response that are significantly modulated throughout the epoch. The model extracted 2 occipital states (state 2 and state 5) with identical spatial maps and ER profiles. The average power spectral density (PSD) plots related to the two states present a 10 Hz peak, which we then associate with alpha activity. These states correspond to state 2 for the 6 states inference presented in the paper.

State 10 embeds the theta prefrontal state that peaks around 200 ms and resembles the theta prefrontal state that we observe in the 6 states inference.

States 3 and 8 present an M300 temporal profile. While state 3 shows a broad frontoparietal activation, state 8 seems to recruit more specifically the sensorimotor regions. However, the temporal characteristics and the spectral content of these states are indistinguishable.

Lastly, state 11 is significantly suppressed early after stimulus onset and it shows upper frontal beta activity. This beta suppression is what we also observe in the 6 states inference in state 3, which, instead, showed mostly beta activity in the sensorimotor cortex.

The task-relevant states detected in the 12 states inference closely resemble the task-relevant states that we reported in our paper, which makes our networks replicable and demonstrates the model reliability. Additionally, we observe two interesting aspects. First, the occurrence of 2 or more states sharing several spatio-spectral features (such as the two occipital states) is a drawback that we risk encountering when increasing the number of states to infer. Secondly, we observe an interesting phenomenon for which our M300 state now seems to split across two states, one specific to prefrontal/temporal functions (resembling the M300 state in the 6 states inference) and one specific to the sensorimotor network. This split might mirror the distinction of two functions (perceptual and motor processing). However, the analysis of these two states is non-trivial because they seem the share temporal and spectral properties.

Lastly, this analysis shows a limitation of this method related to the stochasticity of the inference. By running the model several times, the results are not 100% replicable, and some states might differ in spatio-spectral traits, such as state 11 in the 12 states inference (superior frontal beta) and state 3 in the 6 states inference (sensorimotor beta).

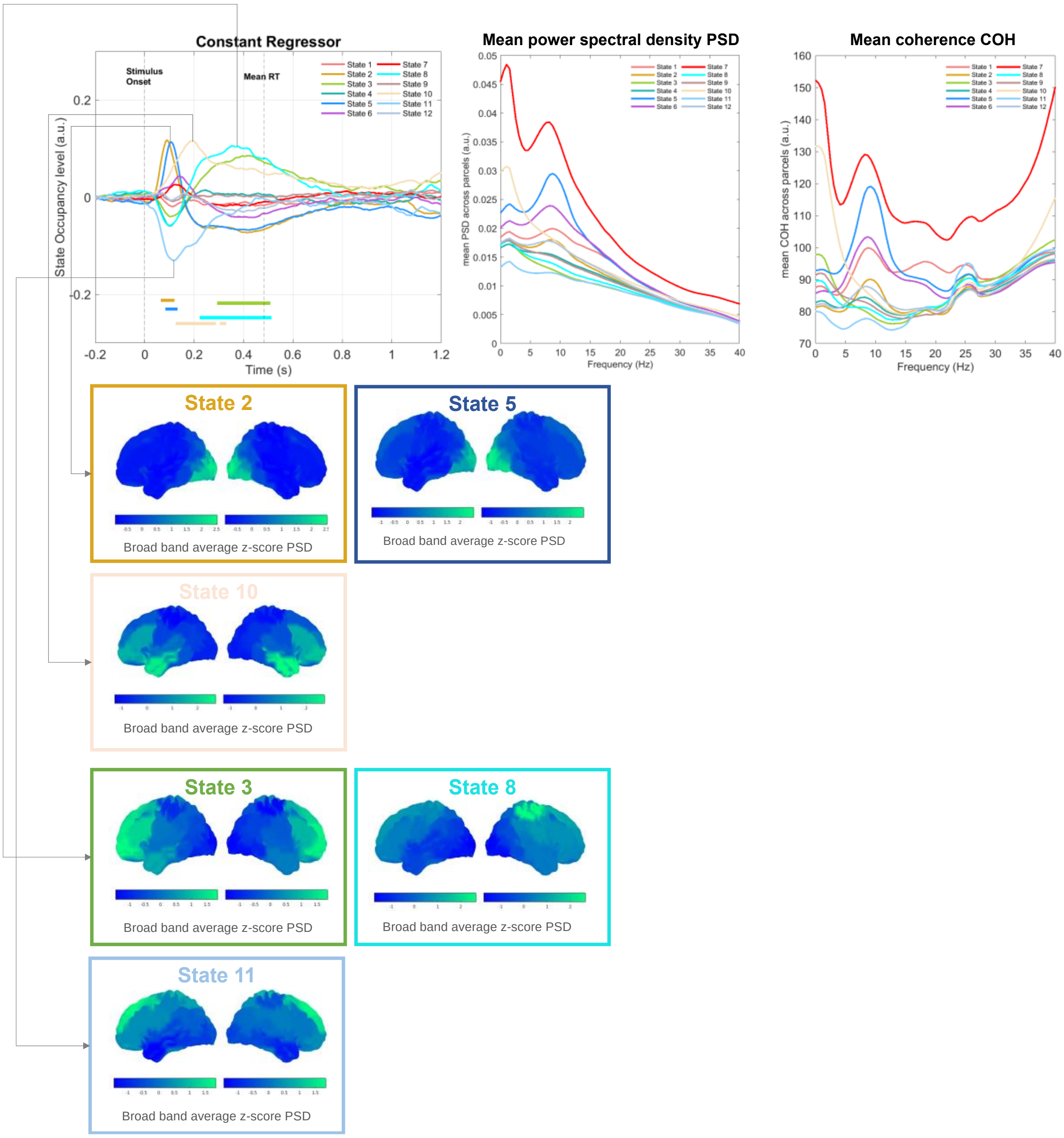

Figure S3. Main results from the 12 states inference. The top left figure reports the average response activation for all 12 states in one epoch (constant regressor of the GLM analysis). Here, we observe 5 states with remarked modulation of their evoked response after stimulus presentation. On the right, the two graphs report the mean power spectral density and phase-coherence for each state, averaged across subjects and regions/connections (second and third plot, respectively). Below, we report the spatial distribution of the z-score power spectral density over the brain, for the relevant states.

3. Temporal characteristics

The temporal characteristics, or temporal properties, are parameters that measures the states' behavior in the time domain. They are computed starting from the Viterbi path, a single time series in which each time point is associated with the most likely state to occur[1]. The temporal characteristics are: the fractional occupancy (FO) that measures the portion of time occupied by one state, the lifetime (LT) that represents the average activation time of a state, and the interval time (IT) that represents the average time window between two consecutive activations of a specific state. These quantities can be computed over the whole concatenated data or per trial.

Over the concatenated data – general descriptors

Considering the fractional occupancy (FO), states 1, 2, and 3 are activated each for about 18% of the time, and state 6 occurs 14% of the time. Instead, state 4 is activated significantly less than all the other states (Wilcoxon rank-sum test, p_value <0.005) with a FO of 11%, and state 5 occurs significantly more frequently than all the other states, with a FO of 22% (Wilcoxon rank-sum test, p_value <0.005). The average lifetime (LT) across all states is 93 ms. States 1, 2, 3, and 6 are activated for approximately 74, 84, 80, and 79 ms, respectively. Instead, states 4 and 5 show significantly longer LTs than the rest of the states: 123, and 116 ms (Wilcoxon rank-sum test, p_value<0.005), respectively. The interval time of states 1, 2, 3, and 5 are 416, 397, 383, and 506 ms, respectively. Instead, the interval time of state 4 is about 1 second, which is significantly longer than all the other states (Wilcoxon rank-sum test, p_value<0.005), and state 6 also appears less frequently than states 1, 2, 3, and 5, with an IT of 506 ms. The temporal characteristics of these states are consistent with the literature considering resting-state and task data [2,3]. All the p values are corrected for multiple comparisons using an FDR function [4].

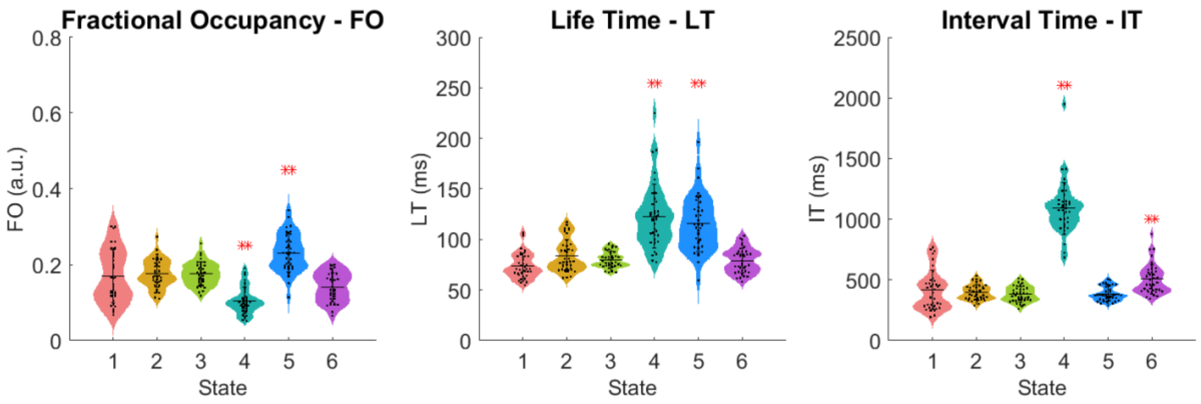

Figure S4 Temporal characteristics extracted from the Viterbi path of continuous data. From left, the fractional occupancy (FO) is evenly distributed across the 6 states, with an average FO per state around 16%. The life time (LT) varies across state, with very short states (1,2,3, and 6) with life times around 60 ms and long states such as 4 and 5 with a LT around 100 ms. The interval time (IT) of state 4 is over 1 sec, whereas the other states occur every 500 ms on average. ** Wilcoxon rank-sum test, p_value<0.005. All the p_values are FDR corrected to solve the multiple comparisons issue.

Per paradigm condition

Following, we computed the LT and IT per paradigm condition. Starting from the Viterbi path, each parameter was extracted per state, per trial, and per subject, and then averaged across trials per subject and paradigm condition. Supplementary Figure S4 reports the statewise distribution of each parameter over subjects per paradigm condition. The LT and the IT of state 5 become longer with increasing WM load and in target as

compared to distractor trials. These results correspond to the results from the evoked-response analysis of the states' time courses. The latter revealed for state 5 an increased occupancy level with increasing WM load and in target as compared to distractor trials (Figure 6 main manuscript). Instead, the event-related responses of states 2 and 3 display significant task-related modulations which do not find a corresponding significant variation in temporal properties. This suggests that, for states 2 and 3, the sustained changes in evoked response results from the average across trials, as also demonstrated by Quinn[5].

This analysis represents a first step towards a trial-based analysis, which is one of the future developments of this work. Additionally, the evaluation of the temporal properties can be crucial when investigating the cognitive impairment in neurological pathologies and their effect on the network dynamics (work under development).

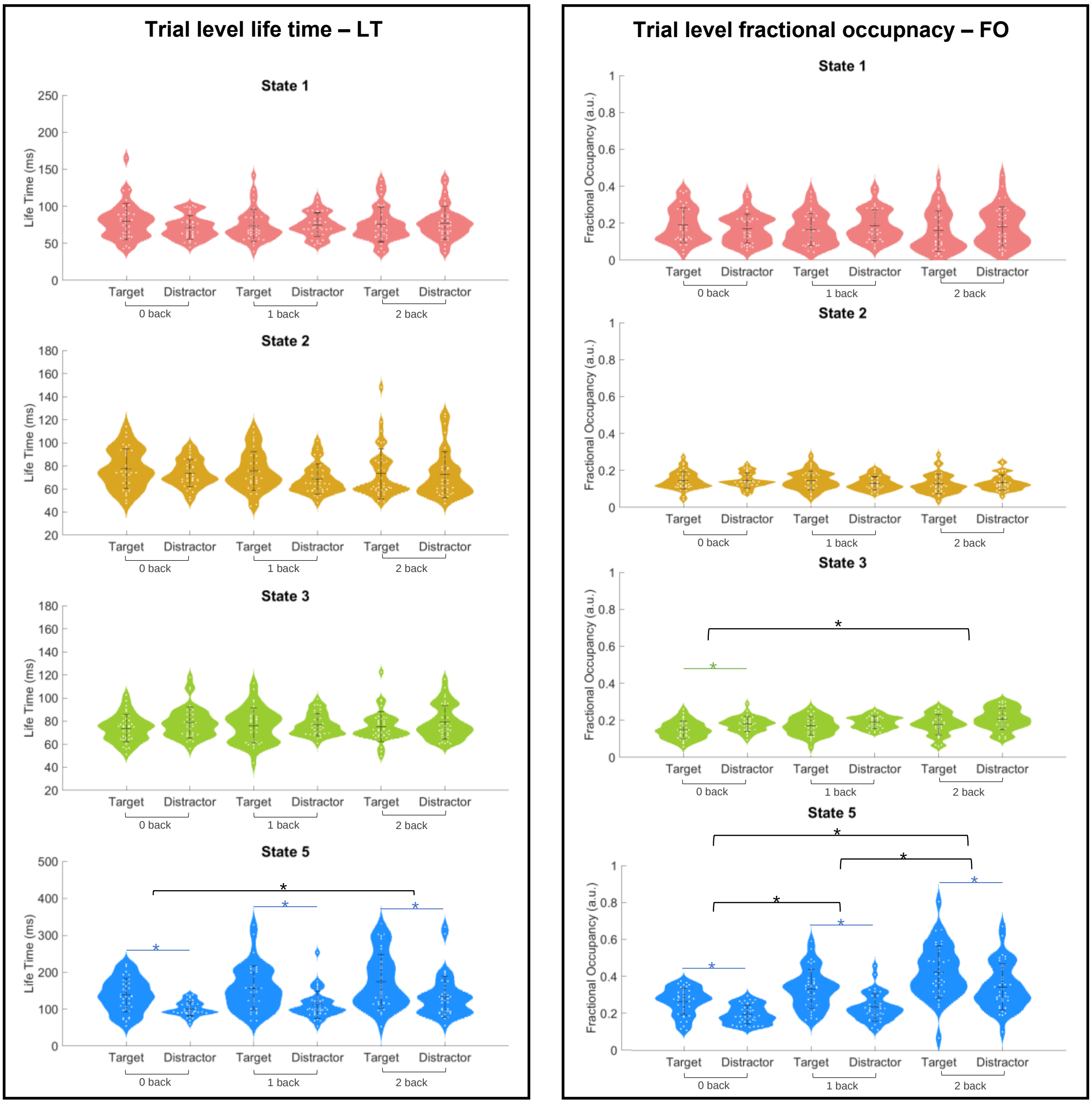

Figure S5. Temporal properties of the task-relevant states. On the left the lifetime (LT), and on the right the fractional occupancy (FO). The temporal characteristics are computed for each paradigm condition, separately to assess their modulation with respect to WM load and target versus distractor. The comparisons are carried out via non-parametric Wilcoxson's rank-sum test, *p_value<0.05. All results are corrected for multiple comparison via FDR correction[4].

4. States Description

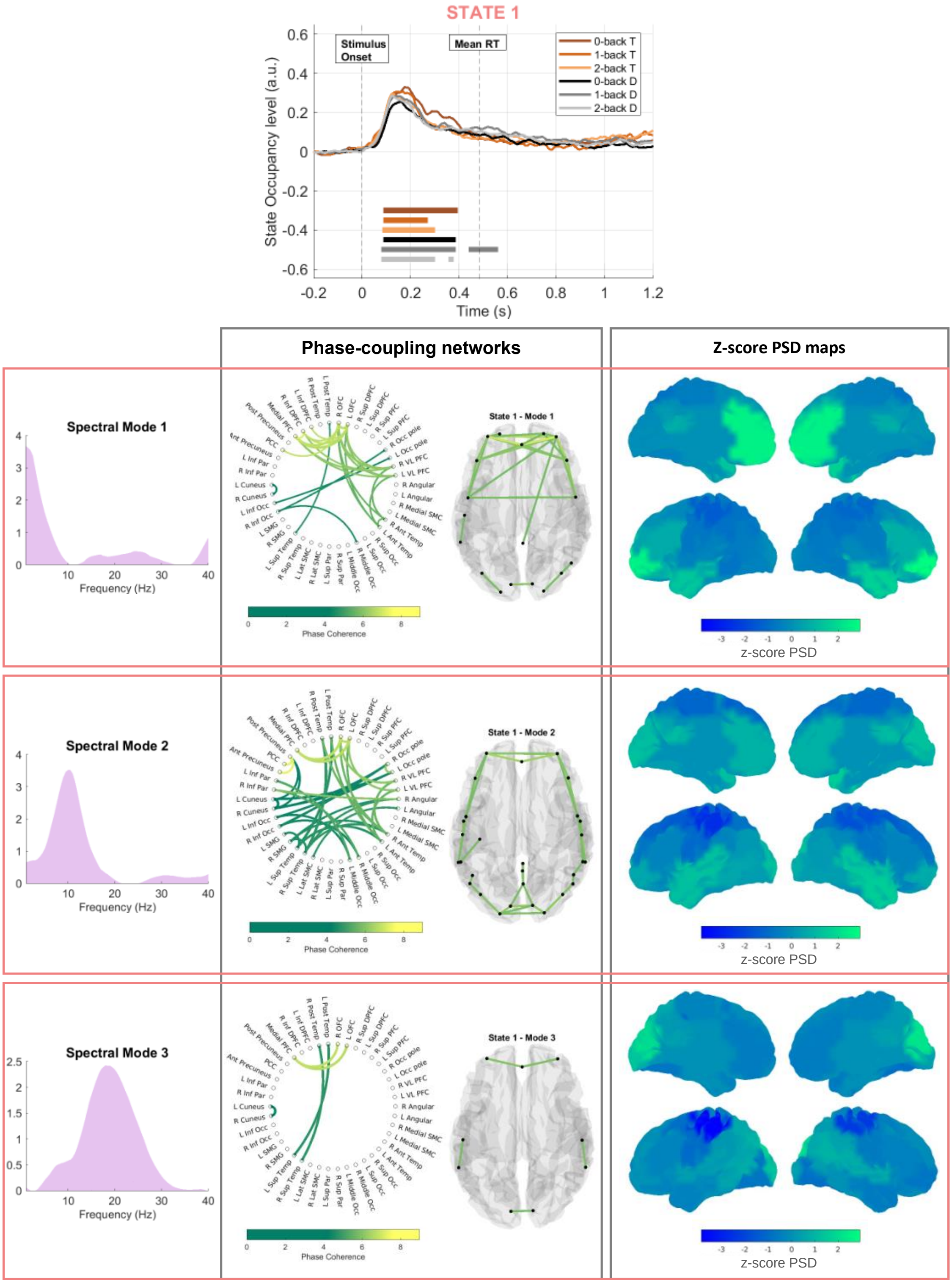

Figure S6 – **State 1** - On top: the task-evoked occupancy level of the state for all the paradigm conditions separately. In the table, the rows consider all the profiles referred to the same spectral mode; the three spectral modes are reported in the first column. The second column shows the connectivity networks with the circular graphs and the brain glasses, and the third column shows the PSD distributions over the brain.

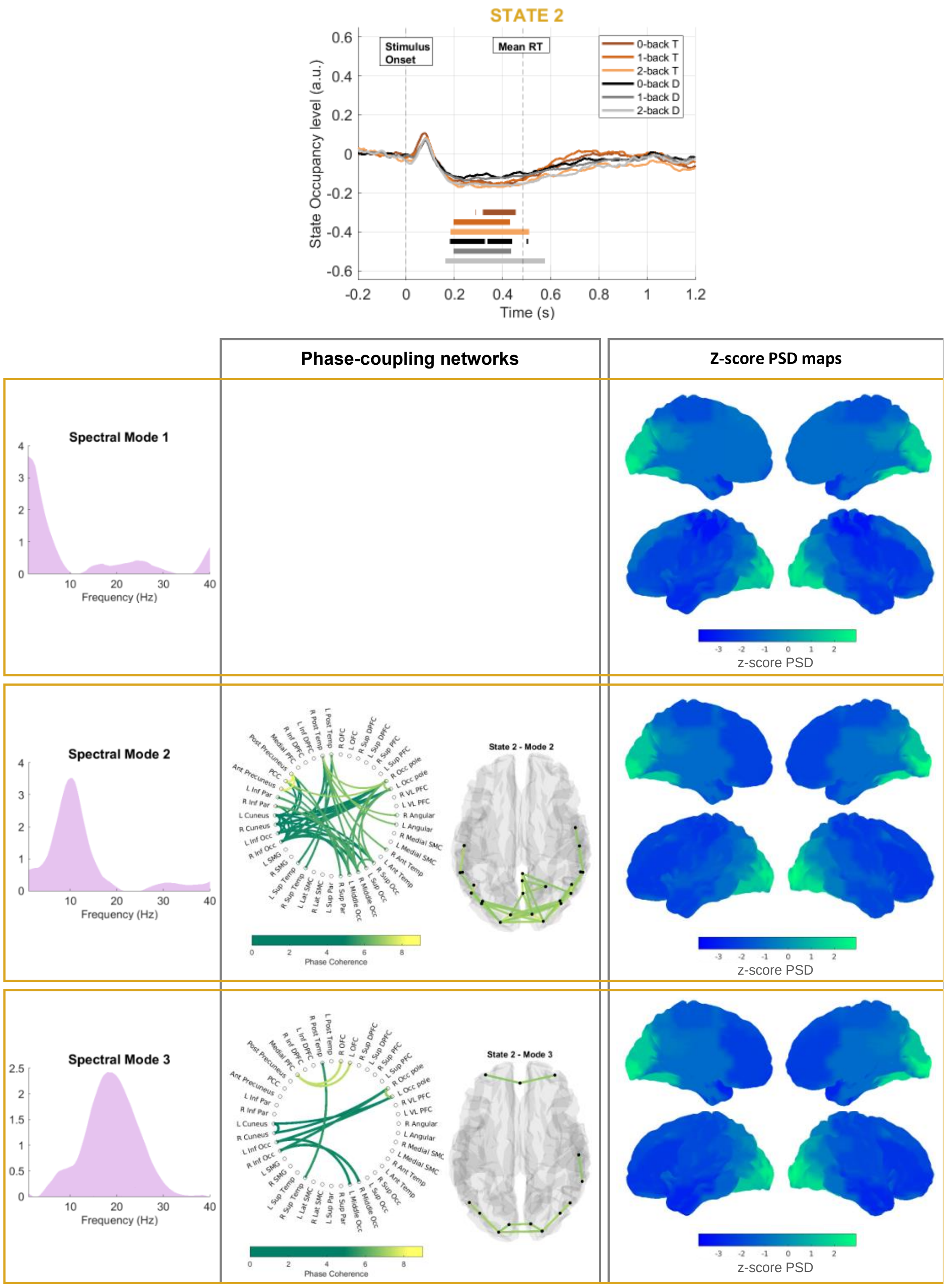

Figure S7 – **State 2** - On top: the task-evoked occupancy level of the state for all the paradigm conditions separately. In the table, the rows consider all the profiles referred to the same spectral mode; the three spectral modes are reported in the first column. The second column shows the connectivity networks with the circular graphs and the brain glasses, and the third column shows the PSD distributions over the brain. The empty box in the connectivity networks column shows that no connections survived thresholding for the connectivity network referred to spectral mode 1.

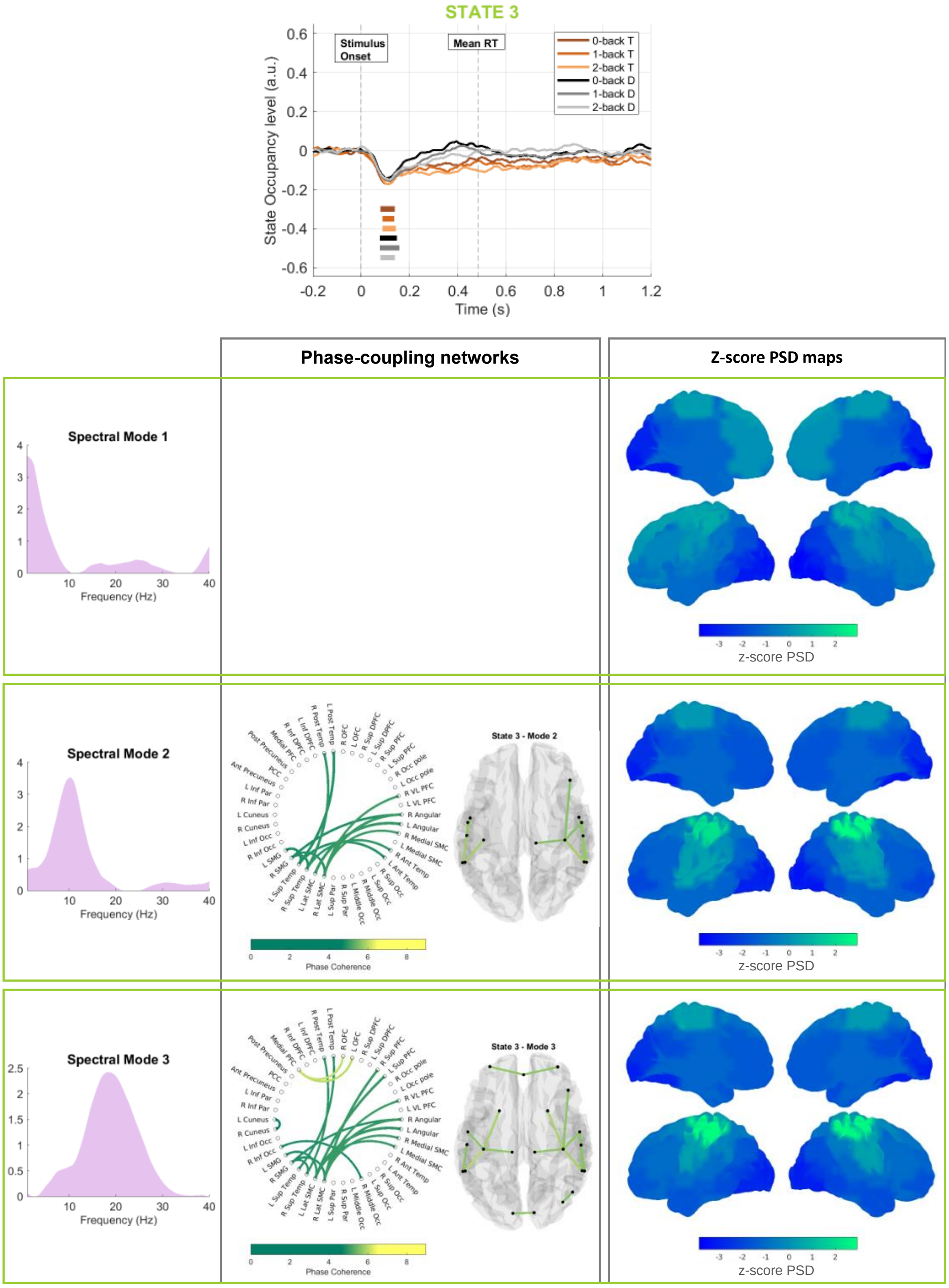

Figure S8 – State 3 - On top: the task-evoked occupancy level of the state for all the paradigm conditions separately. In the table, the rows consider all the profiles referred to the same spectral mode; the three spectral modes are reported in the first column. The second column shows the connectivity networks with the circular graphs and the brain glasses, and the third column shows the PSD distributions over the brain. The empty box in the connectivity networks column shows that no connections survived thresholding for the connectivity network referred to spectral mode 1.

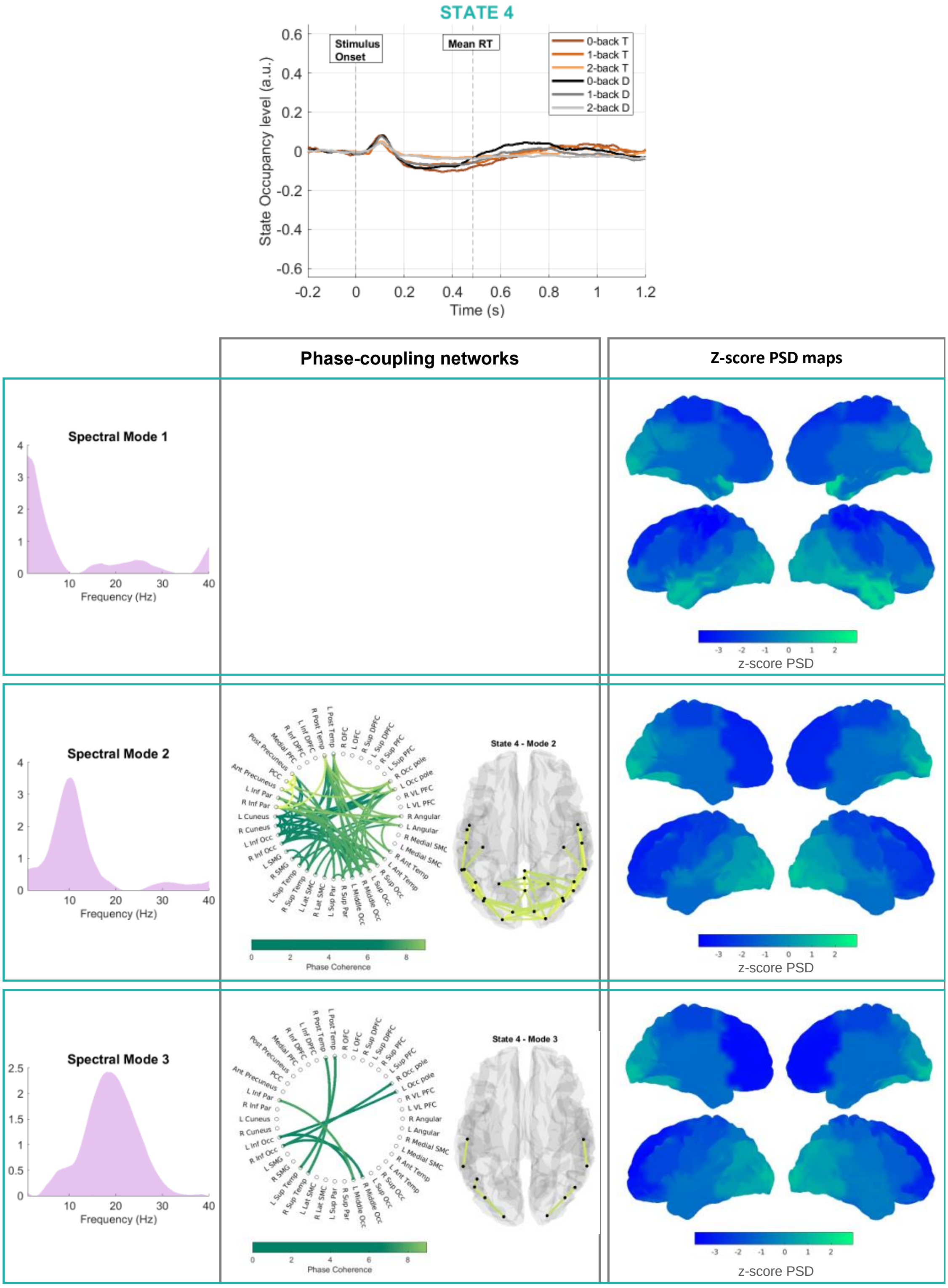

Figure S9 – State 4 - On top: the task-evoked occupancy level of the state for all the paradigm conditions separately. In the table, the rows consider all the profiles referred to the same spectral mode; the three spectral modes are reported in the first column. The second column shows the connectivity networks with the circular graphs and the brain glasses, and the third column shows the PSD distributions over the brain. The empty box in the connectivity networks column shows that no connections survived thresholding for the connectivity network referred to spectral mode 1.

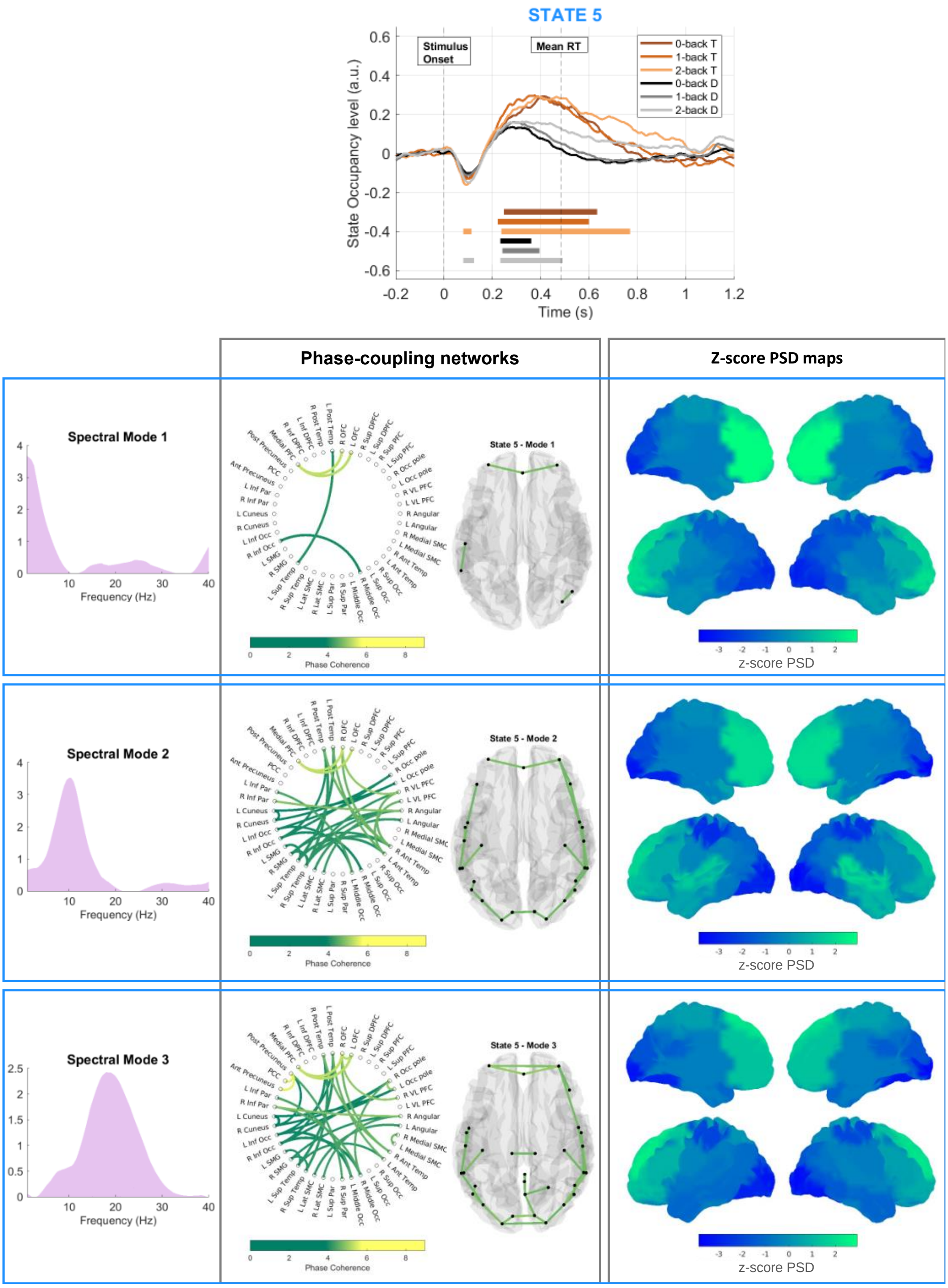

Figure S10 – State 5 - On top: the task-evoked occupancy level of the state for all the paradigm conditions separately. In the table, the rows consider all the profiles referred to the same spectral mode; the three spectral modes are reported in the first column. The second column shows the connectivity networks with the circular graphs and the brain glasses, and the third column shows the PSD distributions over the brain.

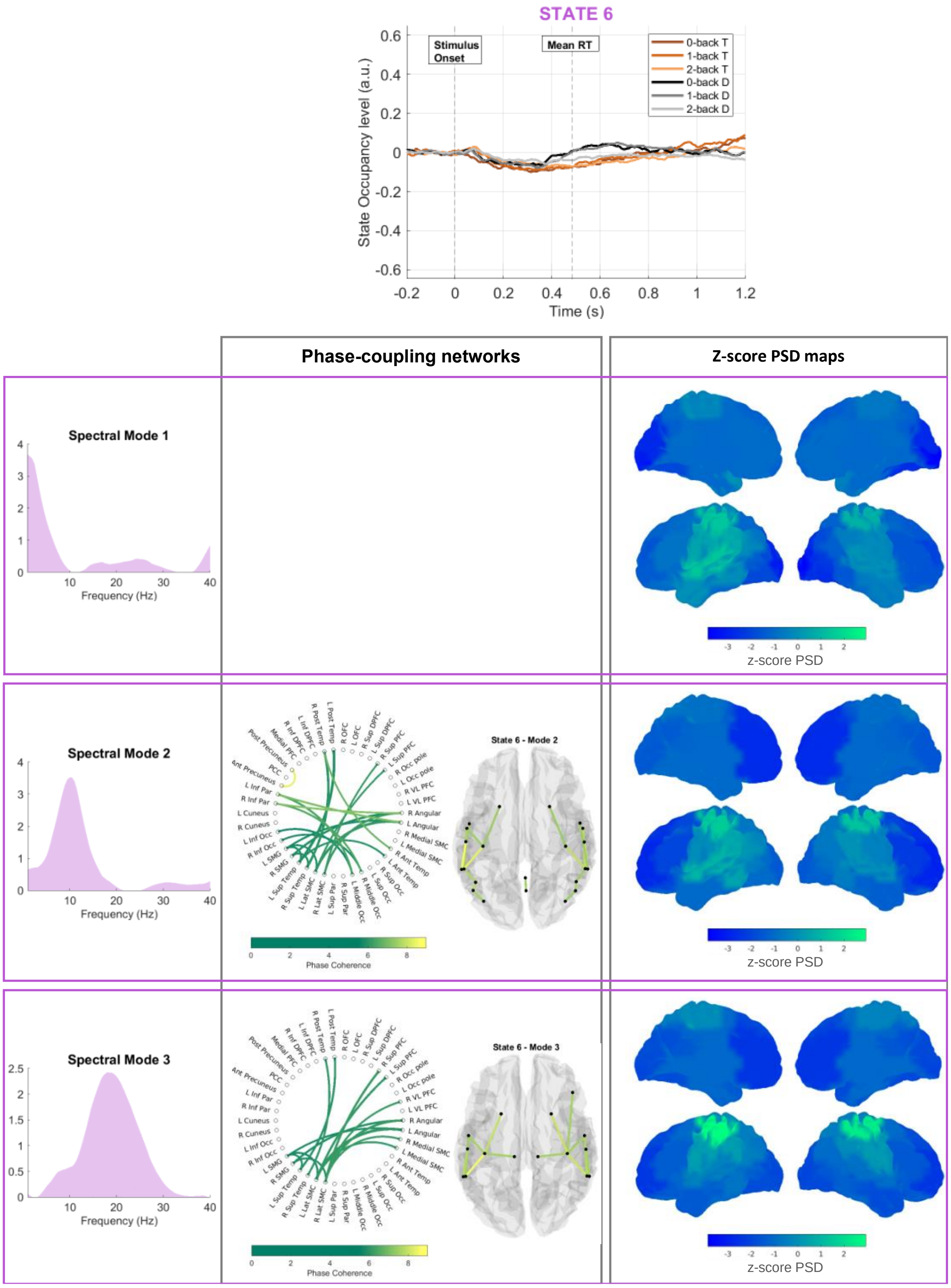

Figure S11 – State 6 - On top: the task-evoked occupancy level of the state for all the paradigm conditions separately. In the table, the rows consider all the profiles referred to the same spectral mode; the three spectral modes are reported in the first column. The second column shows the connectivity networks with the circular graphs and the brain glasses, and the third column shows the PSD distributions over the brain. The empty box in the connectivity networks column shows that no connections survived thresholding for the connectivity network referred to spectral mode 1.

5. Spectral Decomposition

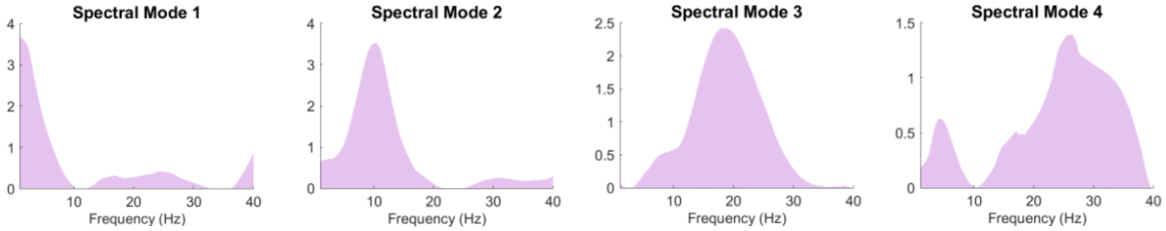

Figure S12 Data-driven frequency bands in which we describe the states frequency content. Spectral mode 1 is associated with the low frequencies, hence, the theta and delta conventional bands. Spectral mode 2 is associated with the alpha band, and spectral mode 3 with the beta band.

Supplementary References

1. Rabiner, L. R. A tutorial on Hidden Markob Models and selected applications in speech recognition. *Proceedings of the IEEE* vol. 77 257–286 (1989).
2. Quinn, A. J. *et al.* Task-evoked dynamic network analysis through Hidden Markov Modeling. *Front. Neurosci.* **12**, 1–17 (2018).
3. Baker, A. P. *et al.* Fast transient networks in spontaneous human brain activity. *Elife* **2014**, 1–18 (2014).
4. Benjamini, Y. & Hochberg, Y. Controlling the False Discovery Rate: A Practical and Powerful Approach to Multiple Testing. *J. R. Stat. Soc. Ser. B* **57**, 289–300 (1995).
5. Quinn, A. J. *et al.* Unpacking Transient Event Dynamics in Electrophysiological Power Spectra. *Brain Topogr.* **32**, 1020–1034 (2019).
