# Supplementary material for: A data-driven network decomposition of the temporal, spatial, and spectral dynamics underpinning visual-verbal working memory processes
Source: Commun Biol. 2023 Oct 23;6:1079. doi: 10.1038/s42003-023-05448-z (PMC10593846; doi:10.1038/s42003-023-05448-z)
Supplement: Supplementary file 3 — Description of Additional Supplementary Data [file 42003_2023_5448_MOESM3_ESM.docx]

**Description of Additional Supplementary Files**

**File name:** Supplementary Data 1

**Description:** The source data behind the graphs displaying the spatial maps. We report the spectral modes (Supplementary Figure S7), and the source data behind the plots of the power spectral density distributions for all states and all spectral modes (Figure 2 and 3 in the paper, and supplementary Figures S6 to S11) Each tab is named after the figure that results from the provided data.

**File name:** Supplementary Data 2

**Description:** The source data behind the graphs displaying the phase-coupling networks for all the states and spectral modes (Figures 2 and 5, supplementary Figures S6 to S11).

**File name:** Supplementary Data 3

**Description:** The source data behind the graphs of the states time courses, as result of the GLM analysis of the states posterior probabilities (Figures 2, 3, 4 and supplementary Figures S6 to S11).

**File name:** Supplementary Data 4

**Description:** The source data behind the figures regarding the methods and the supplementary materials. We include the data related to the 12 states inference (Figure S3), and the data behind the plots composing Figure 1 and 6 related to the methods.
