# Supplementary material for: A data-driven network decomposition of the temporal, spatial, and spectral dynamics underpinning visual-verbal working memory processes
Source: Commun Biol. 2023 Oct 23;6:1079. doi: 10.1038/s42003-023-05448-z (PMC10593846; doi:10.1038/s42003-023-05448-z)
Supplement: Supplementary file 8 — Reporting summary [file 42003_2023_5448_MOESM8_ESM.pdf]

nature portfolio

Corresponding author(s): Chiara Rossi
Jeroen Van Schependom

Last updated by author(s): 7/10/2023

Reporting Summary

Nature Portfolio wishes to improve the reproducibility of the work that we publish. This form provides structure for consistency and transparency in reporting. For further information on Nature Portfolio policies, see our Editorial Policies and the Editorial Policy Checklist.

Statistics

For all statistical analyses, confirm that the following items are present in the figure legend, table legend, main text, or Methods section.

n/a	Confirmed	
☐	☒	The exact sample size (n) for each experimental group/condition, given as a discrete number and unit of measurement
☐	☒	A statement on whether measurements were taken from distinct samples or whether the same sample was measured repeatedly
☐	☒	The statistical test(s) used AND whether they are one- or two-sided<br>*Only common tests should be described solely by name; describe more complex techniques in the Methods section.*
☐	☒	A description of all covariates tested
☐	☒	A description of any assumptions or corrections, such as tests of normality and adjustment for multiple comparisons
☐	☒	A full description of the statistical parameters including central tendency (e.g. means) or other basic estimates (e.g. regression coefficient) AND variation (e.g. standard deviation) or associated estimates of uncertainty (e.g. confidence intervals)
☐	☒	For null hypothesis testing, the test statistic (e.g. F, t, r) with confidence intervals, effect sizes, degrees of freedom and P value noted<br>*Give P values as exact values whenever suitable.*
☐	☒	For Bayesian analysis, information on the choice of priors and Markov chain Monte Carlo settings
☐	☒	For hierarchical and complex designs, identification of the appropriate level for tests and full reporting of outcomes
☒	☐	Estimates of effect sizes (e.g. Cohen's d, Pearson's r), indicating how they were calculated

Our web collection on statistics for biologists contains articles on many of the points above.

Software and code

Policy information about availability of computer code

Data collection	We collected the MEG data with two MEG systems: Neuromag VectorViewTM system (13 subjects), and then the updated NeuromagTM TRIUX system (MEGIN Oy, Croton Healthcare, Helsinki, Finland) (25 subjects)
Data analysis	We performed all the analyses in the MATLAB 2020b environment. In particular, we used the OSL package (version updated to April 2021) which can be found here https://github.com/OHBA-analysis/osl .

For manuscripts utilizing custom algorithms or software that are central to the research but not yet described in published literature, software must be made available to editors and reviewers. We strongly encourage code deposition in a community repository (e.g. GitHub). See the Nature Portfolio guidelines for submitting code & software for further information.

Data

Policy information about availability of data

All manuscripts must include a data availability statement. This statement should provide the following information, where applicable:

- Accession codes, unique identifiers, or web links for publicly available datasets
- A description of any restrictions on data availability
- For clinical datasets or third party data, please ensure that the statement adheres to our policy

The data for this study are not publicly available because of legal concerns. Researchers interested in a collaboration on these data are welcome to contact the senior authors. Analysis scripts are available upon request from the corresponding author.

Human research participants

Policy information about studies involving human research participants and Sex and Gender in Research.

Reporting on sex and gender	Sex was not considered during the study design. In fact, the sample size was not computed to assess the effect of sex on the neurophysiological response, since we expect sex to be irrelevant to the neurophysiological activity underlying working memory. We presented the demographics (age and education) of the dataset for the male and female groups, separately.
Population characteristics	The dataset includes 38 healthy subjects, among which 23 are female and 15 are male. The age of the male group is 49.4± 6.9 (mean age and standard deviation) and the education level of the same group is 14.9 ± 3.1 (mean years of education and standard deviation). For the female group, the mean age is 42.7± 11 (years and standard deviation) and the education level is 14.7± 3.3 (mean years of education and standard deviation).
Recruitment	The healthy subjects considered in this study are part of a larger data collection that included healthy subjects and patients with multiple sclerosis. The data collection of both groups took place simultaneously. An advertisement for the data collection was sent out via the communication platform of the university hospital in Brussels (Universitair Ziekenheuis Brussel, Jette, Brussels) and the National MS center in Melsbroek (Belgium). No self-selection biases occurred in the recruitment procedure.
Ethics oversight	National MS Center Melsbroek and University Hospital Brussels (Commissie Medische Ethiek UZ Brussel, B.U.N. 143201423263, 2015/11)

Note that full information on the approval of the study protocol must also be provided in the manuscript.

Field-specific reporting

Please select the one below that is the best fit for your research. If you are not sure, read the appropriate sections before making your selection.

☒ Life sciences ☐ Behavioural & social sciences ☐ Ecological, evolutionary & environmental sciences

For a reference copy of the document with all sections, see nature.com/documents/nr-reporting-summary-flat.pdf

Life sciences study design

All studies must disclose on these points even when the disclosure is negative.

Sample size	The data used in this study were collected during a bigger data collection recruiting healthy controls and patients with multiple sclerosis. The sample size was calculated considering the effect size of the difference in P300 peaks of the neurophysiological responses between the two groups. In this paper, we provide an analysis only using the healthy control data.
Data exclusions	Although 50 healthy subjects were recruited, we only consider 38 healthy subjects for the following reasons: 1 subject did not perform the task (hence we do not have the MEG data), 11 subjects were excluded for very low quality of MEG data as determined by a visual data quality check before analysis.
Replication	We did not replicated the analysis on a different dataset. However, the model we used (TDE-HMM) is publicly available (see above data analysis section) and it has already been used to analyse different dataset (Quinn et al 2018, Vidaurre et al 2018, etc..).
Randomization	We did not need to randomize the subjects because we only consider one group of healthy subject on which the analysis is performed.
Blinding	Blinding was not relevant to our study because we only considered one group of healthy controls and subjects were not allocated to different subgroups.

Reporting for specific materials, systems and methods

We require information from authors about some types of materials, experimental systems and methods used in many studies. Here, indicate whether each material, system or method listed is relevant to your study. If you are not sure if a list item applies to your research, read the appropriate section before selecting a response.

March 2021

Materials & experimental systems

n/a	Involved in the study
☒	☐ Antibodies
☒	☐ Eukaryotic cell lines
☒	☐ Palaeontology and archaeology
☒	☐ Animals and other organisms
☒	☐ Clinical data
☒	☐ Dual use research of concern

Methods

n/a	Involved in the study
☒	☐ ChIP-seq
☒	☐ Flow cytometry
☐	☒ MRI-based neuroimaging

Magnetic resonance imaging

Experimental design

Design type

The MRI data were only structural. Instead, the MEG data were acquired during an n-back task.

Design specifications

As the MRI data were only structural, no experimental design is defined. Instead, we defined the experimental design for the MEG data acquisitions. The following description of the paradigm design is identical for all the recordings (hence, for all the subjects) Twelve blocks of 20 letters (stimuli) each were presented pseudo-randomly, four for each paradigm condition (0-back, 1-back, 2-back). The total number of target trials (when subject needs to press the button) is 25, 23, and 28, for the 0, 1, and 2-back conditions, respectively; the rest of the trials are referred to as distractors.

Behavioral performance measures

Again, we did not measure behavioral data during the MRI. Instead, we measured the reaction time of response during the MEG recordings. The reaction time is defined as the time window between the stimulus onset (captured by a diode located below the screen) and the moment of button press.

Acquisition

Imaging type(s)

structural

Field strength

3T

Sequence & imaging parameters

The 3D MR images were T1-weighted, a longitudinal MRI and the subjects were in HFS position. The scan used an echo pulse sequence gradient with Echo sequence TE 2.3 s, the recording parameters were TR = 4.939 ms, flipping angle 8, field of view 230 × 230 mm2, number of sagital slices 310, resulting in a 0.53 by 0.53 by 0.5 mm3 resolution (voxel). The slice size was 1 and the space between size 0.5.

Area of acquisition

whole brain scan

Diffusion MRI ☐ Used ☒ Not used

Preprocessing

Preprocessing software

We used FSL to preprocess the MRI imges.

Normalization

The data were not normalized because the structural images were only use to inform the source-reconstruction process of the MEG data.

Normalization template

As no normalization process was carried out on the MRI data, no normalization template was used either.

Noise and artifact removal

We did not perform noise and artifact removal during the course of this study, but we only used preprocessed MRI data. The preprocessing was conducted as explained in S. Jain et al. NeuroImage: Clinical 2015.

Volume censoring

No volume censoring was performed in this work because the main analysis was not carried out on MRI data but rather on MEG data.

Statistical modeling & inference

Model type and settings

No statistical analysis was performed on the MRI data. We applied the TDE-HMM technique to analyse MEG source-reconstructed data. We inferred 6 brain states and performed a two-level GLM analysis on the states' time courses to study their activation throughout the epoch of interest.

Effect(s) tested

We did not test for any effect on the MRI data.

Specify type of analysis: ☒ Whole brain ☐ ROI-based ☐ Both

Statistic type for inference
(See Eklund et al. 2016)

We didn't perform any statistical analysis on MRI data. Regarding the MEG data, we ran permutations to test for states activation over time in the different paradigm conditions (0, 1, and 2 back target and distractor trials). For behavioral data, we used a ranksum test to observe the differences in reaction time and accuracy of response between paradigm conditions (0, 1, 2 back).

Correction

We didn't perform any statistical analysis on MRI data. All the statistical analyses on MEG data were corrected for multiple comparisons by either FDR correction (after the ranksum test for simple analyses such as the reaction time and the accuracy of performance) or by maximum statistics after permutation testing.

Models & analysis

n/a	Involved in the study
☐	☒ Functional and/or effective connectivity
☒	☐ Graph analysis
☒	☐ Multivariate modeling or predictive analysis

Functional and/or effective connectivity

We did not perform any analysis on the MRI data. We performed a data-driven dynamic functional connectivity analysis on MEG data by applying the time delay embedded - hidden Markov model as described in Vidaurre et al. Nature Communications 2018. We let the model infer 6 brain states. The spectral information of each state is extracted by running a multitaper on the subject-specific MEG data weighted by the posterior probability of each state (the time course of each state). From this multitaper, we can then build the phase-coupling network of the state by computing the phase-coherence between pairs of regions, and a spatial map as the mean power spectral density distribution over the brain. The spatial map and the phase-coherence network define the functional connectivity in this work.
